# Transarterial Embolization With N-Butyl-2-Cyanoacrylate for Rapidly Enlarging Distal Anterior Choroidal Artery Aneurysm Associated With Moyamoya Disease: A Case Report

**DOI:** 10.7759/cureus.65340

**Published:** 2024-07-25

**Authors:** Shimpei Tsuboki, Takafumi Mitsutake, Keisuke Kadooka, Michihiro Tanaka

**Affiliations:** 1 Department of Neuroendovascular Surgery, Kameda Medical Center, Kamogawa, JPN; 2 Department of Neurosurgery, Kameda Medical Center, Kamogawa, JPN

**Keywords:** anterior choroidal artery, moyamoya disease (mmd), endovascular treatment (evt), transarterial embolization (tae), peripheral aneurysm

## Abstract

Peripheral aneurysms associated with moyamoya disease, particularly those originating from the anterior choroidal artery, often have a poor prognosis and are typically managed with endovascular treatments. Comprehensive imaging diagnostics and anatomical expertise are critical in minimizing ischemic complications during treatment. We present a case of a 55-year-old woman with a rapidly enlarging distal anterior choroidal artery aneurysm identified during an intracerebral hemorrhage associated with moyamoya disease. The patient underwent super-selective embolization using N-butyl-2-cyanoacrylate (NBCA) during the chronic phase, resulting in a favorable outcome. Detailed intraoperative imaging was essential in guiding the treatment and mitigating risks.

## Introduction

Moyamoya disease is associated with cerebral aneurysms in 3-15% of cases [1-4], with approximately 40% of these being peripheral aneurysms [3]. Reports suggest a high risk of rupture for aneurysms originating in the anterior choroidal artery [5]. Additionally, in moyamoya disease, perforating arteries and choroidal arteries can serve as the primary vessels supplying the cortex [3], necessitating careful attention to ischemic complications during endovascular treatment.

This report presents a case of a rapidly enlarging distal anterior choroidal artery aneurysm treated with transarterial embolization (TAE) using N-butyl-2-cyanoacrylate (NBCA), highlighting the significance of detailed intraoperative imaging.

## Case presentation

**Figure 1:**
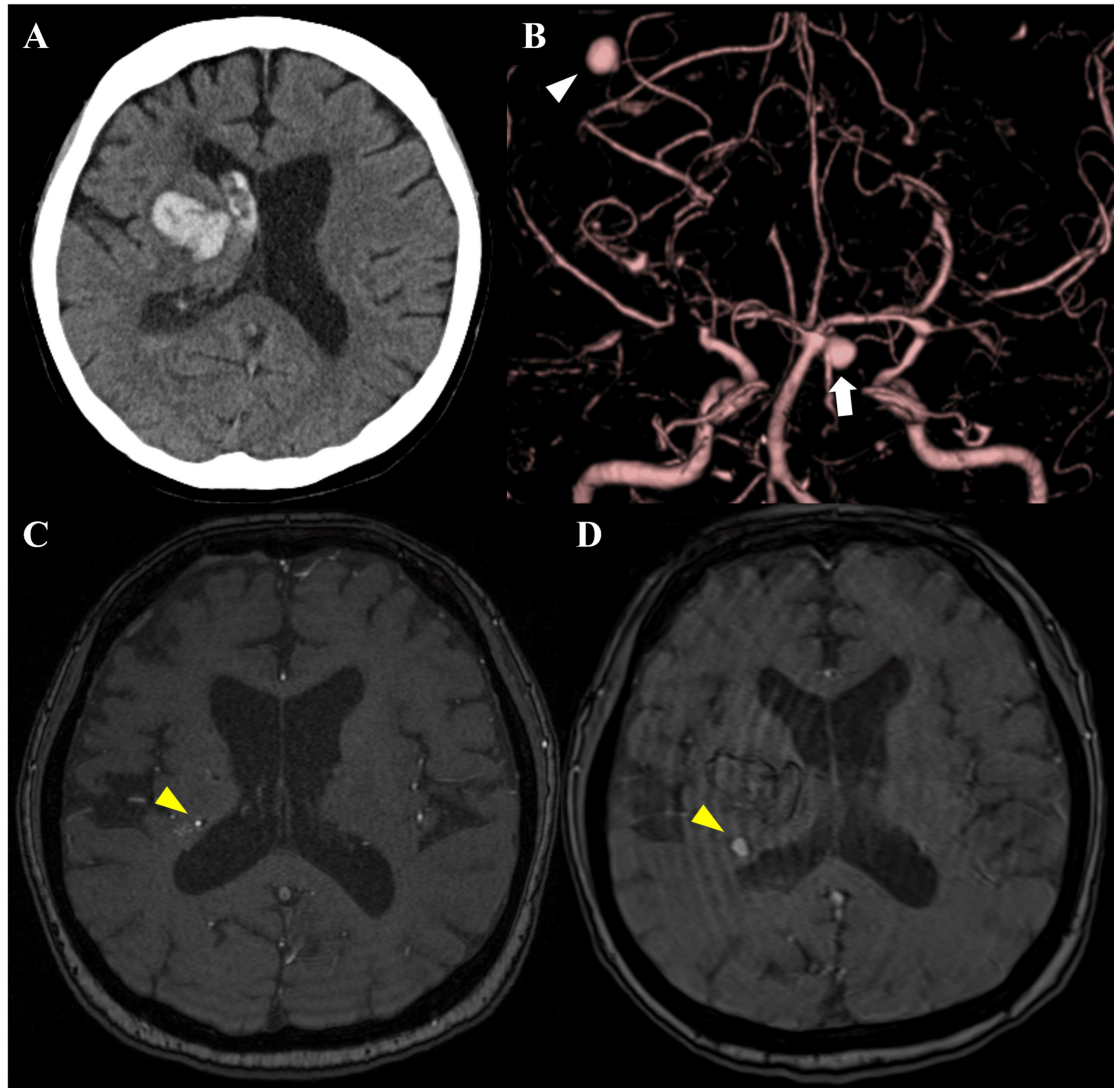
Radiographic images on admission. A head computed tomography (CT) scan in the emergency room revealed a right thalamic and intraventricular hemorrhage (A). Three-dimensional CT angiography identified two aneurysms: one in the left basilar-superior cerebellar artery (white arrow) and another in the right cerebral hemisphere behind the hematoma (white arrowhead) (B). A magnetic resonance angiography (MRA) performed two months prior to her presentation showed a small aneurysm of about 1 mm in the right corona radiata (C), and an MRA performed the day after presentation disclosed the aneurysm had grown to approximately 5 mm (yellow arrowheads) (D).

A 55-year-old female with a history of schizophrenia, for which she was taking quetiapine, had been regularly monitored for moyamoya disease with an MRI at her local clinic. She was brought to our emergency room after suddenly experiencing left-sided hemiparesis. A head computed tomography (CT) scan revealed a right thalamic hemorrhage (Figure 1A), and three-dimensional CT angiography identified an aneurysm in the left basilar-superior cerebellar artery and another aneurysm behind the hematoma (Figure 1B). Comparing a magnetic resonance angiography (MRA) performed two months prior to her presentation with one performed at our hospital, we observed rapid growth of the aneurysm (Figures 1C, 1D).

**Figure 2:**
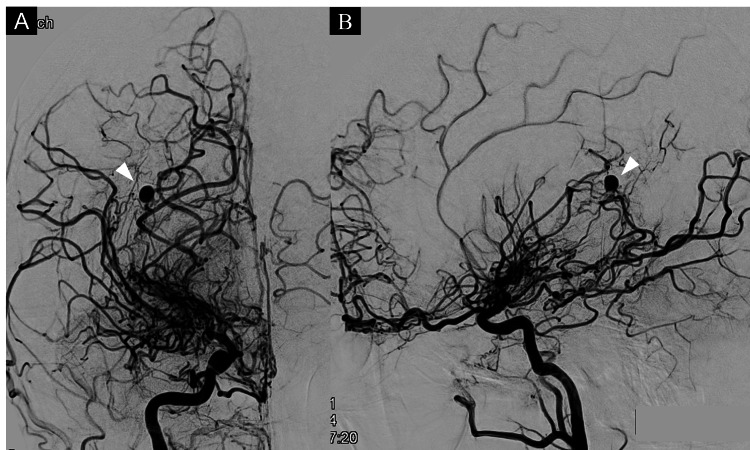
Angiography in the acute phase of intracerebral hemorrhage. Frontal (A) and lateral (B) views of the acute-phase right internal carotid artery angiography showed an aneurysm in the peripheral anterior choroidal artery (white arrowheads).

**Figure 3:**
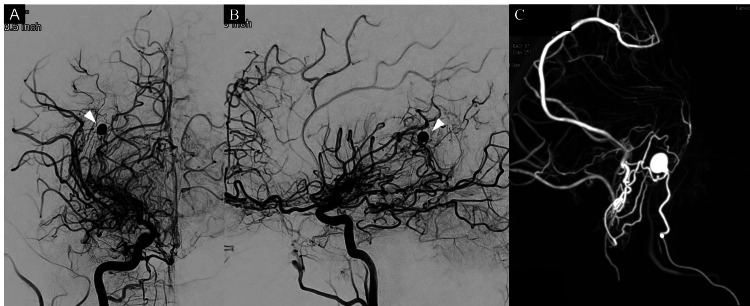
Angiography prior to embolization. Frontal (A) and lateral (B) views of the right internal carotid artery angiography showed an aneurysm in the peripheral anterior choroidal artery (white arrowheads). Compared to the angiography performed in the acute phase, the aneurysm had neither shrunk nor disappeared. Super-selective contrast cone-beam computed tomography revealed a 6 mm aneurysm and ventriculofugal collateral supply to the right posterior parietal lobe (C).

Acute-phase cerebral angiography revealed that the aneurysm behind the hematoma was located at the distal segment of the capsulothalamic artery, which branched from the cisternal segment of the anterior choroidal artery (Figure 2). We opted for conservative treatment during the acute phase. One month after onset, under general anesthesia, we performed embolization for the left basilar-superior cerebellar artery aneurysm and follow-up right internal carotid artery angiography (Figures 3A, 3B). The anterior choroidal artery aneurysm did not shrink or disappear and was also embolized simultaneously.

**Figure 4:**
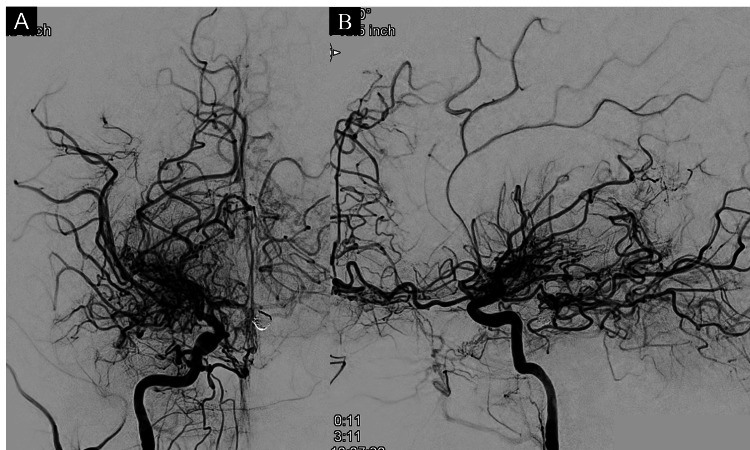
Angiography after embolization. Frontal (A) and lateral (B) views of the right internal carotid artery angiography after the procedure confirmed complete obliteration of the aneurysm.

A 6-Fr Emboy guiding catheter (90 cm STR, Cordis Co., Miami Lakes, FL) was advanced into the right internal carotid artery. Using a 0.008-inch CHIKAI microguidewire (ASAHI Intecc Co., Tokyo, Japan), a flow-directed Magic 1.5 Fr. microcatheter (Balt, Montmorency, France) was navigated to the proximal medullary course of the anterior choroidal artery. Super-selective angiography under cone-beam computed tomography (CBCT) revealed a 6 mm aneurysm and ventriculofugal collateral supply to the right posterior parietal lobe (Figure 3C). A mixture of approximately 10% NBCA and lipiodol was injected, adequately filling the aneurysm while preserving ventriculofugal cortico-medullary circulation. Control angiography confirmed complete obliteration of the aneurysm (Figure 4).

Postoperatively, the patient experienced transient worsening of her left hemiparesis, but MRI showed no new ischemic changes. After continued rehabilitation, she was transferred to another facility for further rehabilitation with a modified Rankin Scale (mRS) score of 4 one month post-procedure, and she was discharged home with an mRS score of 4 four months after the procedure.

## Discussion

This case report presents a distal anterior choroidal artery aneurysm associated with moyamoya disease effectively treated with NBCA embolization following a detailed radiographic evaluation. Distal anterior choroidal artery aneurysms in moyamoya disease are rare, and there is no consensus on the optimal treatment approach. Treatment options primarily include observation, direct surgery, and endovascular treatment, although advancements in imaging technology and devices have led to an increase in reports of endovascular treatments. Previous reports indicate that conservative management of hemorrhagic cases of moyamoya disease results in a 30% rebleed rate [5]. While some cases have shown aneurysm resolution through hemodynamic load reduction following revascularization surgery [6], there is also a report of aneurysm rupture 10 years post-revascularization [7]. Open surgical management of aneurysms has the advantage of hematoma removal but can involve cortical incision, potentially sacrificing brain parenchyma and collateral circulation depending on the location of the hematoma and aneurysm. There have been 11 reported cases of endovascular treatment for these aneurysms (Table 1). Treatment approaches vary depending on the location and presentation of the aneurysm. It is crucial to consider whether the aneurysm is a true aneurysm or a pseudoaneurysm when determining the treatment method. One report indicated that pseudoaneurysms were present in one out of five cases. Consequently, when endovascular treatment is possible, parent artery occlusion should be considered, with seven out of 11 reported cases involving parent artery occlusion. Regarding embolic materials used for endovascular treatment, coils were used in six cases, NBCA in three cases, and Onyx (Medtronic, Dublin, Ireland) and Glubran (GEM, Viareggio, Italy) in one case each. In cases where the parent artery was preserved, all were treated with coil embolization. When using liquid embolic agents like NBCA, Onyx, or Glubran, attention must be paid to the risk of ischemic complications.

**Table 1:** Reported cases of distal anterior choroidal artery aneurysm associated with moyamoya disease treated with endovascular treatment. M: male; F: female; ICH: intracerebral hemorrhage; IVH: intraventricular hemorrhage; SAH: subarachnoid hemorrhage; NBCA: N-butyl-2-cyanoacrylate; PAO: parent artery occlusion.

Author	Age (years)	Sex	Hemorrhage	Embolic materials	Type of embolization
Sugiura et al. [8]	47	M	ICH	Coil	Selective coil
Kim et al. [9]	43	F	IVH	NBCA	PAO
Choulakian et al. [10]	35	F	IVH	NBCA	PAO
Yang et al. [11]	38	F	IVH	Glubran	PAO
Yang et al. [11]	56	F	Unruptured	Coil	Selective coil
Okamura et al. [5]	39	F	IVH	Coil	Selective coil
Murakami et al. [12]	32	F	ICH	NBCA	PAO
Schmalz et al. [13]	40	M	SAH	Coil	PAO
Liu et al. [14]	11	M	IVH	Onyx	PAO
Zhang et al. [15]	Young	F	IVH	Coil	PAO
Crowe et al. [16]	1	M	ICH	Coil	Selective coil
Present case	55	F	Unruptured	NBCA	PAO

The anterior choroidal artery is divided into the cisternal and plexal segments. Embolization is generally safe if it is accomplished beyond the plexal segment, which is corresponding to the entrance of the inferior horn of the lateral ventricle. In this case, the aneurysm was located in the capsulothalamic artery, a perforator that usually branches from the most distal part of the cisternal segment. Embolization of the capsulothalamic artery carries a high risk of ischemic complications in the thalamus and posterior limb of the internal capsule. In this case, the surrounding parenchyma had already been injured by the hemorrhage, so the potential for new symptoms was considered limited. The patient was fully informed of the risks before the procedure.

During the procedure, we aimed to navigate the catheter as distally as possible to minimize the segment of embolization and associated symptoms. In moyamoya disease, with the chronic occlusion of the terminal internal carotid artery, moyamoya vessels proliferate, and perforating arteries and choroidal arteries may anastomose with ventriculofugal arteries to supply the cortex [4]. Generally, cortical ischemia due to proximal occlusion is rare due to leptomeningeal anastomosis, but when using liquid embolic agents, there is a risk of distal migration leading to cortical ischemia. Therefore, careful attention is required during embolization.

In this case, cortical arteries were visualized during microcatheter angiography, prompting us to take a contrast-enhanced CBCT from the microcatheter for careful image confirmation. By determining and sharing the exact location of the anastomosis and the planned embolization endpoint with the team, we were able to achieve a safe embolization.

This report has some limitations. First, the association between antipsychotic drugs and stroke needs to be considered. While the use of antipsychotic drugs has been linked to ischemic stroke [17], there have been no reports connecting them to the occurrence of moyamoya disease or aneurysms. In this case, as it involves hemorrhagic stroke, the association appears to be minimal; however, the potential relationship between stroke and antipsychotic medications should not be overlooked. Additionally, this report presents a single case and does not definitively establish the safety or efficacy of embolization for distal anterior choroidal artery aneurysms. Further accumulation of reports is needed in the future.

## Conclusions

We report a case of TAE with NBCA for a distal anterior choroidal artery aneurysm with anastomosis to cortical arteries. Given the pathology of moyamoya disease, there is a risk of cortical infarction due to the inadvertent migration of liquid embolic agents during endovascular treatment. Detailed intraoperative imaging analysis based on high-resolution CBCT is crucial to ensure safe and effective embolization.
